# Relationship between Changes in Body Fat and a Decline of Renal Function in the Elderly

**DOI:** 10.1371/journal.pone.0084052

**Published:** 2014-01-16

**Authors:** Se Won Oh, Shin Young Ahn, Xu Jianwei, Ki Woong Kim, Sejoong Kim, Ki Young Na, Dong Wan Chae, Suhnggwon Kim, Ho Jun Chin

**Affiliations:** 1 Department of Internal Medicine, Eulji General Hospital, Eulji University College of Medicine, Seoul, Korea; 2 Department of Internal Medicine, Seoul National University Bundang Hospital, Kyeong-Kido, Korea; 3 Department of Psychiatry, Seoul National University College of Medicine, Seoul, Korea; 4 Department of Internal Medicine, Seoul National University, Seoul, Korea; 5 Renal Institute, Seoul National University Medical Research Center, Seoul, Korea; University Medical Center Utrecht, Netherlands

## Abstract

Obesity is a risk factor for chronic kidney disease, and its prevalence among the elderly is increasing. We investigated the effects of changes in body fat percentage (BFP) on the longitudinal changes in the estimated glomerular filtration rate (eGFR) in the elderly. This prospective cohort study included 390 participants aged >65 years who underwent bioelectrical impedance analysis at baseline and follow-up as a part of the Korean Longitudinal Study on Health and Aging. After a median follow-up period of 5.3 years, BFP was significantly higher than that at the start point (*P*<0.05). Participants who had the largest increase in BFP had the highest BMI and waist circumference (WC) (*P<*0.001). The highest tertile had the highest white blood cell count and erythrocyte sedimentation rate, incidence of rapid progression, and decline in eGFR >25% (*P≤*0.017, *P* = 0.025, *P* = 0.005, respectively). The lowest tertile had the lowest triglyceride and highest high-density lipoprotein levels (*P*<0.05). The adjusted decline rate in eGFR was correlated with a change in BFP (*P* = 0.039), but not with that in BMI or WC. The highest tertile had a 4.875-fold increase in the risk for rapid progression to a decline in eGFR (95% CI: 1.366–17.397) and a 4.931-fold decrease in the risk to a decline in eGFR>25% (95% CI: 1.617–15.037), when compared with the lowest tertile. In subgroup analysis, the incidence of renal outcomes was significantly increased according to the increase in BFP in patients with lower eGFR (*P≤*0.010). A change in BFP may be associated with inflammation and dyslipidemia development, and longitudinal changes in body fat are related to a decrease in eGFR in the elderly.

## Introduction

Obesity is a well-known risk factor for cardiovascular disease (CVD) and mortality in the general population [1], [2]. Several epidemiologic studies have reported that obesity is related to the development of chronic kidney disease (CKD) and end-stage renal disease (ESRD) [3], [4], [5], [6] .Previous studies have shown that a high body mass index (BMI) is a strong, independent risk factor for ESRD in a large population of Asian patients as well as amongst Caucasians [3], [4]. A BMI of ≥25 kg/m^2^ at the age of 20 years was associated with a 3-fold increase in the risk for CKD, and a BMI of ≥30 kg/m^2^ and ≥35 kg/m^2^ amongst men and women, respectively, was associated with a 3- to 4-fold increase in the risk of CKD [5]. Nevertheless, recent studies indicated that BMI is paradoxically associated with a lower risk of mortality in the elderly [7], [8], [9], [10], [11], [12], [13]. The relative risk for all-cause and cardiovascular mortality decreased significantly with increasing age in a population with a higher BMI [1], [8]. The paradoxical relationship was also noted in CKD. Higher BMI was associated with improved survival in patients with CKD and dialysis [14], [15]. The prevalence of CVD and CKD is the highest and rapidly increases in the elderly, as does the prevalence of obesity [16], [17]. It is not yet known whether obesity is less harmful or even protective in the elderly, and whether BMI is an appropriate marker of obesity for the prediction of adverse outcomes in this population.

Longitudinal cohort studies indicate that BMI and body weight reduce slightly or do not change at all in the elderly, despite the loss of height, bone, and muscle during the aging process [18], [19], [20]. Aging is associated with considerable changes in body composition. From 20 to 70 years of age, fat-free mass reduces by 40%, and maximal body fat is attained at the age of 60–70 years [18], [19], [20]. Therefore, the change of body composition could be a useful marker of obesity.

The aim of this study was to investigate the effects of body fat change on the longitudinal change of estimated glomerular filtration rate (eGFR) in the elderly. We also assessed the association between body fat change, inflammation, and lipid profile.

## Materials and Methods

### Study participants

**Figure 1:**
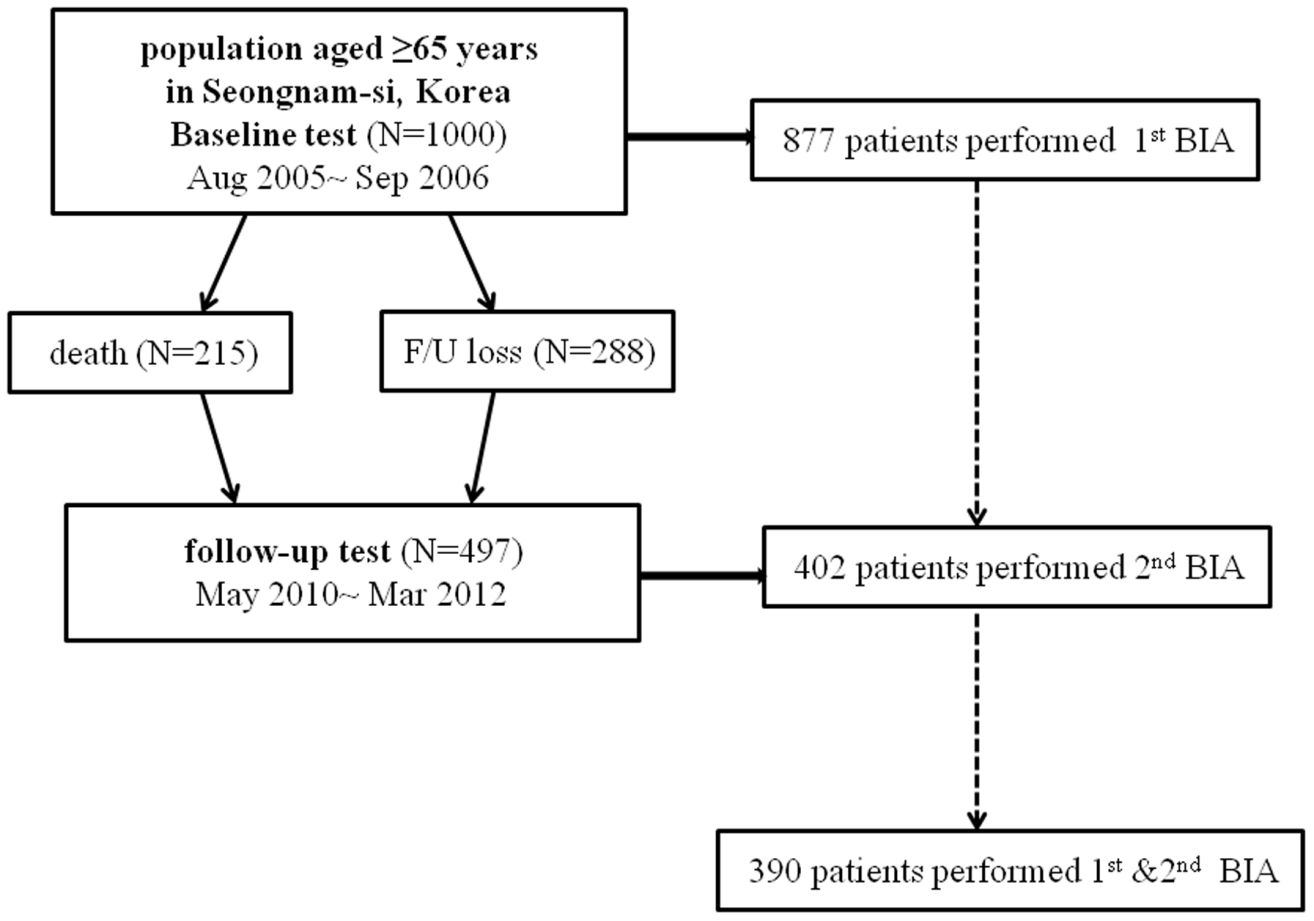
Identification of study participants.

This was a population-based, prospective cohort study comprising Korean men and women aged ≥65 years, residing in Seongnam-si, a satellite city in Seoul, Korea. This study was a part of the Korean Longitudinal Study on Health and Aging (KLoSHA), and the study design has been previously described in detail [21]. The baseline study and second phase of KLoSHA commenced in September 2005 and May 2010, respectively. Among the 1000 participants who underwent baseline tests, 215 died and 288 were lost during the mean follow-up period of 5.3 years. Three-hundred-and-ninety of the 877 participants who underwent baseline bioelectrical impedance analysis (BIA) tests (Inbody 720, biospace Co., Seoul, Korea) were followed up with the BIA tests (Figure 1). This study protocol was reviewed and approved by the institutional review board of the Seoul National University Bundang Hospital (B-0508/023-003) with patients written consent given. The study was conducted in accordance with the Declaration of Helsinki.

### Measurements and definitions

Investigated clinical parameters included age; sex; a history of alcohol intake, smoking, hypertension, diabetes mellitus; and cerebrovascular accident (CVA). Systolic blood pressure (SBP) and diastolic blood pressure (DBP) were measured after the participants had rested for at least 3 minutes. The serum creatinine level was measured using the alkaline picrate Jaffe kinetic method with an automatic analyzer (Toshiba 200FR; Tokyo, Japan). Serum creatinine levels were calibrated to an assay traceable on an isotope dilution mass spectrometry device (Roche diagnostics). GFR was calculated using the CKD-epidemiology collaboration equation [22]. Patients fulfilling one of the following criteria were defined as being hypertensive: SBP ≥140 mmHg, DBP ≥90 mmHg, or use of antihypertensive medication irrespective of BP. Diabetes mellitus was defined as a fasting glucose level of ≥126 mg/dL or the use of hypoglycemic agents. Waist-to-hip-ratio (WHR) was defined as the ratio of the circumference of the waist to that of the hip. Proteinuria was defined as albumin ≥1+. The annual decline rate of eGFR was calculated as follows: change of eGFR [(eGFR at follow up – eGFR at baseline)/follow-up period (years)]. Rapid progression in renal dyfunction was defined as a decline of eGFR ≥4 ml/min/1.73 m^2^/year [23]. A history of exercise was defined as regular exercise more than once a week. BIA was measured using Inbody 720 (Biospace, Seoul, Korea).

### Statistical analysis

All analyses were performed using SPSS software (SPSS version 17.0; Chicago, IL, USA). Data are presented as mean ± standard deviations (SD) for continuous variables and as proportions for categorical variables. Differences were analyzed using the χ^2^ test for categorical variables and the student *t*-test or analysis of variance for non-categorical variables. Differences between measurements taken at baseline and follow-up were compared using a paired *t*-test. Participants were divided into 3 groups according to tertiles of changes in body fat percentage (BFP). Analysis of covariance (ANCOVA) was used to adjust independent factors related to eGFR in order to determine the difference in the annual decline of eGFR. The unadjusted relative risks for a >25% decline in eGFR were calculated using logistic regression analysis, and adjustments were made for age, gender, and other variables that had a *P* value<0.05 in univariate analyses. Values of *P*<0.05 were considered statistically significant.

## Results

### Changes in total body fat and loss of body weight

Table 1 shows the values of the physical and nutritional parameters at baseline. Study participants had a higher body mass, height, BMI, and WHR (*P*<0.05) than non-study participants. In addition, study participants had a higher serum protein level, hemoglobin level, and eGFR (*P*<0.05) than non-study participants. However, the excluded participants did not differ from study population with regard to total body fat.

**Table 1:** Findings for physical and nutritional parameters at baseline and follow-up.

	Baseline study		Follow-up study
	Total (N = 877)	Study participants (N = 390)	Study participants (N = 390)
Body weight (kg)	57.6±10.9	61.0±9.9*	60.1±10.7†
Height (cm)	156.8±9.3	158.5±8.9*	157.7±9.2†
BMI (kg/m^2^)	24.0±3.3	24.2±3.1*	24.1±3.3
WC (cm)	86.8±9.2	86.9±9.2	87.5±8.8
WHR	0.93±0.08	0.93±0.09*	0.93±0.37
Total body fat (kg)	17.2±5.6	17.4±5.3	18.9±6.1†
Total body fat (%)	28.9±7.2	28.4±7.0	31.3±8.0†
eGFR (mL/min/1.73 m^2^)	72.3±17.0	75.8±15.4*	73.1±15.5†
Serum protein (mg/dL)	7.5±0.5	7.5±0.4*	7.2±0.4†
Serum cholesterol (mg/dL)	202.8±37.9	202.7±38.8	187.4±36.2†
Serum TG (mg/dL)	134.7±81.2	141.0±97.3	131.3±77.2†
Serum Hb (g/dL)	13.7±1.5	14.0±1.5*	13.8±1.5†

Total body fat was significantly increased, and body weight, and height reduced in the study participants. However, the body mass index did not change during the follow-up period.

Abbreviations: BMI, body mass index; WC, waist circumference; WHR, waist-to-hip ratio; eGFR, estimated glomerular filtration rate; TG, triglyceride; Hb, hemoglobin.

*P*<0.05, study participants vs. non-study participants among baseline study.

*P*<0.05, baseline study vs. follow-up study among study participants.

After a mean follow-up period of 5.3 years, study participants showed a loss of height (−0.2±12.3 cm) and body weight (−1.0±4.7 kg) (*P*<0.05). Serum protein level, cholesterol level, triglyceride (TG) level, hemoglobin (Hb) level, and eGFR were reduced in the follow-up study (*P*<0.05), but total body fat was significantly increased (1.5±3.3 kg [2.9±4.3%], *P*<0.05). BMI, WC, and WHR did not change significantly. Total body fat was markedly increased despite loss of height and body weight and a decrease in nutritional parameters.

### Correlation between the changes of body fat percentage and the markers of inflammation and metabolic syndrome

Participants were divided into tertiles according to the changes in BFP (BFP at follow-up – BFP at baseline). Median values of BFP change were −0.5 (25–75%, −2.7 to 0.7), 3.2 (2.4 to 3.9), and 6.2 (5.3 to 8.0) in the lowest, middle, and highest tertiles, respectively. At baseline, the patients in different tertiles did not differ in age; sex; SBP; BMI; WC; WHR; or prevalence of hypertension, diabetes mellitus, and CVA. In addition, there was no difference in the eGFR, prevalence of proteinuria, levels of inflammatory markers such as white blood cell count (WBC) and erythrocyte sedimentation rate (ESR), and nutritional markers such as HbA1c and albumin among the tertiles. Only the lipid profiles showed a weak, but significant difference at baseline: the TG levels were higher in the middle tertile than in the lowest tertile (*P* = 0.047) and the high-density lipoprotein (HDL) levels differed significantly between the tertiles (*P* = 0.049) (Table 2).

**Table 2:** Clinical parameters of baseline and follow-up according to the change of body fat percentage.

	Baseline study				Follow-up study			
	Changes of body fat percentage				Changes of body fat percentage			
	1st	2nd	3rd	*P*	1st	2nd	3rd	*P*
Age (yr)	71.3±6.9	70.6±6.0	71.6±6.4	0.429				
Sex (%)	72 (55.4)	69 (53.1)	58 (44.6)	0.188				
SBP (mmHg)	133.2±16.6	132.5±15.3	131.1±18.3	0.611	125.3±16.7	123±15.9	125.6±16.0	0.379
BMI (kg/m^2^)	24.2±2.9	24.4±3.1	24.2±3.4	0.829	23.0±2.8	24.2±3.2	25.1±3.4	<0.001
WC (cm)	85.5±8.8	85.6±8.9	87.9±9.7	0.085	84.4±7.9	86.9±7.7	89.6±8.7	<0.001
WHR	0.91±0.08	0.91±0.09	0.93±0.09	0.247	0.90±0.6	0.90±0.05	0.92±0.05	0.017
eGFR(ml/min/1.73 cm^2^)	75.4±13.9	75.5±15.6	75.5±16.2	0.996	75.2±13.3	73.6±15.7	70.9±16.6	0.075
Proteinuria(%)	10 (7.8)	6 (4.7)	8(6.2)	0.576	6(4.7)	7 (5.5)	8(6.2)	0.867
WBC(×10^3^/µL)	6.1±1.7	6.4±1.6	6.3±1.6	0.183	5.7±1.6	6.1±1.56	6.3±1.7	0.017
ESR (mm/hr)	19.1±13.6	16.5±10.4	18.6±13.4	0.194	12.7±12.1	11.2±9.7	16.1±14.4	0.006
HDL (mg/dL)	63.6±15.4	59.4±14.3	60.0±15.1	0.049	55.5±14.2	52.0±12.2	51.8±13.1	0.043
TG (mg/dL)	124.2±67.3	153.0±105.1	138.2±103.1	0.047	113.7±56.3	141.3±87.0	139.5±84.2	0.007
HbA1c (%)	6.1±0.9	6.1±0.9	6.1±0.8	0.971	6.02±0.70	6.18±0.79	6.25±0.89	0.087
Albumin (g/dL)	4.1±0.2	4.2±0.2	4.1±0.2	0.124	4.4±0.3	4.4±0.2	4.4±0.3	0.549
HTN (%)	87 (66.9)	93 (71.5)	95 (73.1)	0.527				
DM (%)	28 (21.5)	36 (27.7)	34 (26.2)	0.492				
CVA (%)	16 (12.3)	14 (10.8)	12 (9.2)	0.625				
Exercise (%)	100 (76.9)	86 (66.2)	74 (56.9)	0.003				

Measurements taken at follow-up showed marked differences in body composition, inflammation level, and lipid profile amongst tertiles of body fat change, despite no differences at baseline.

Abbreviations: BMI, body mass index; WC, waist circumference; WHR, waist-to-hip ratio; eGFR, estimated glomerular filtration rate; WBC, white blood cell, ESR, erythrocyte sedimentation rate; HDL, high density lipoprotein cholesterol, TG, triglyceride; HTN, hypertension; DM, diabetes mellitus, CVA, cerebrovascular accident.

At follow-up, changes in BFP were significantly correlated with physical parameters. The highest BMI, WC, and WHR were seen in participants who had the largest increase in BFP (*P*≤0.017). In addition, inflammatory markers such as WBC and ESR were highest in the highest tertile (*P*≤0.017). The lowest tertile had the lowest TG level and the highest HDL level (*P* = 0.007 and *P* = 0.043 for TG and HDL levels, respectively). Although there were no differences at baseline, at follow-up, the changes of BFP significantly correlated with the parameters of inflammation and metabolic syndrome. In addition, the change of body fat was significantly associated with regular exercise at baseline (table 2).

### Correlation between annual decline in the estimated GFR and changes in body fat percentage

**Figure 2:**
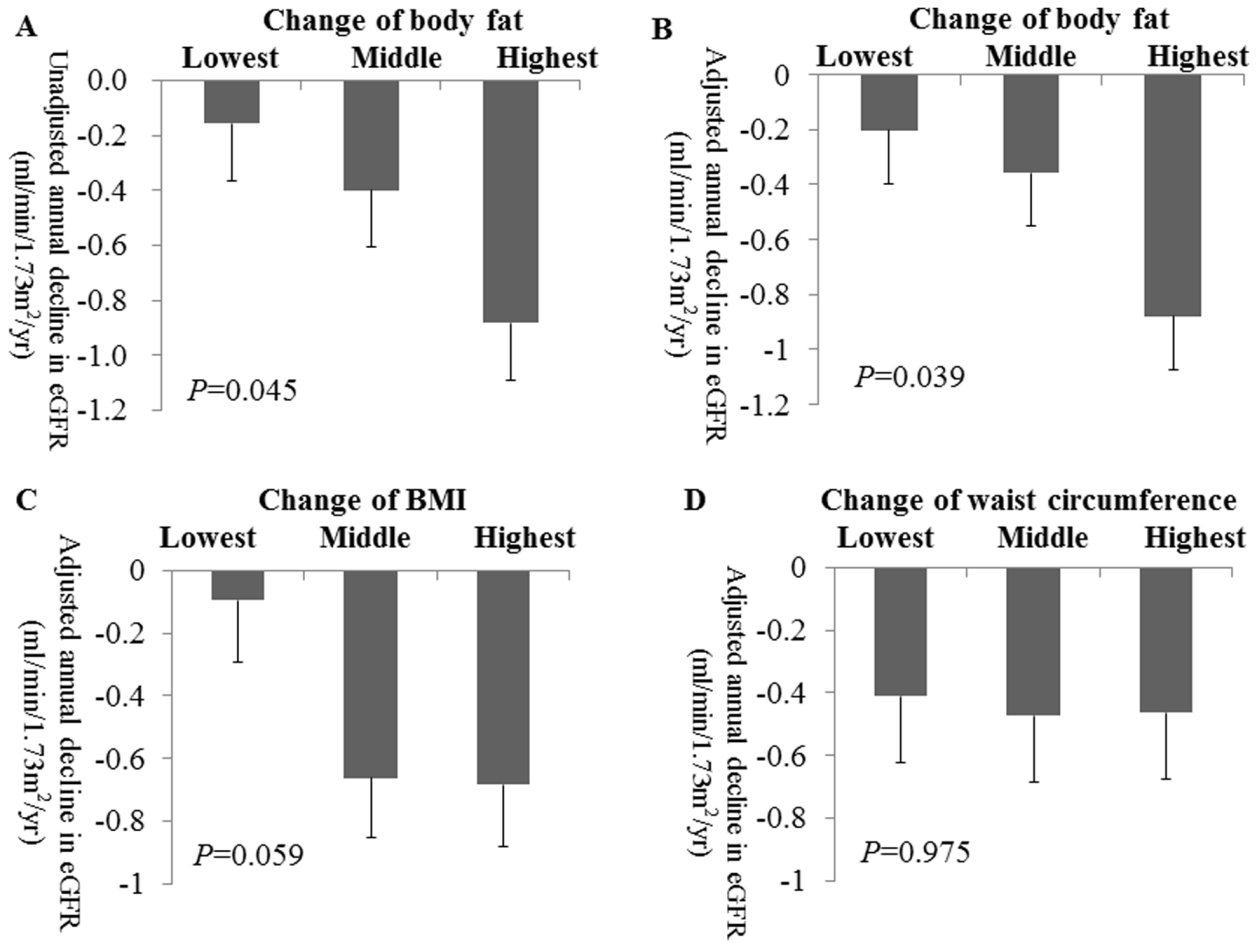
Annual decline in eGFR is correlated with change of body fat. (A) Unadjusted annual decline in eGFR amongst tertiles of change in body fat percentage. (B) Adjusted annual decline in eGFR among tertiles of change in body fat percentage. (C) Adjusted annual decline in eGFR among tertiles of change in BMI. (D) Adjusted annual decline in eGFR among tertiles change of waist circumference. Error bars indicate standard error of mean. Annual decline in eGFR was adjusted using analysis of covariance (ANCOVA) by age; sex; diabetes; hypertension; smoking history; and baseline values of systolic blood pressure, fasting glucose, body mass index, serum creatinine, triglyceride, high-density lipoprotein.

In the study population, eGFR reduced by an average of −0.5±2.3 ml/min/1.73 m^2^/year, and rapid progression occurred in 6.4% of the study participants. We analyzed the difference in the annual decline in eGFR in the tertile groups based on changes of BFP, BMI, and WC, to ascertain the effect of BFP change and other markers of obesity on the decline in renal function. Unadjusted means of the eGFR decline were significantly higher in the highest than in the lowest tertile (*P* = 0.045) (Figure 2A). Adjusted rates of eGFR decline were also higher in the highest tertile (lowest, −0.203 (95% CI: −0.589 to 0.184); middle, −0.359 (95% CI: −0.740 to 0.021); highest, −0.879 (95% CI, −1.260 to −0.499); *P* = 0.039; Figure 2B). There were no significant differences in the adjusted rate of decline in eGFR in the tertile groups based on change of BMI and WC (Figure 2 C,D).

### Decline in estimated GFR in the highest tertile

We investigated the incidence of rapid progression in renal dysfunction among tertiles. Rapid progression in the decline of renal function was most frequently seen in the highest tertile (3.3%, 4.8%, and 11.2% in the lowest, middle, and highest tertile, respectively; *P* = 0.025). Multivariable-adjusted analysis showed that the highest tertile had a 4.875-fold increase in the risk for rapid progression in renal dysfunction when compared with the risk in the lowest tertile (95% CI: 1.366–17.397, Table 3).

**Table 3:** Odds ratio for renal outcome according to the changes of body fat.

Tertile group of changes of body fat percent	Unadjusted (95% CI)	*P*	Age and sex adjusted (95% CI)	*P*	Multivariable-adjusted^a^ (95% CI)	*P*
**Rapid progression** b						
**1st** −0.45(−2.16∼−1.00)	reference	0.035	reference	0.031	reference	0.027
**2nd** 3.13(2.98∼3.29)	1.500 (0.413–5.451)	0.538	1.530 (0.420–5.572)	0.519	1.786 (0.432–7.387)	0.423
**3rd** 7.09 (6.69∼7.50)	3.752 (1.199–11.743)	0.023	3.900 (1.239–12.277)	0.020	4.875 (1.366–17.397)	0.015
**≥25% decline in eGFR**						
**1st** −0.45(−2.16∼−1.00)	reference	0.008	reference	0.009	reference	0.007
**2nd** 3.13(2.98∼3.29)	1.423 (0.440–4.604)	0.556	1.483 (0.457–4.815)	0.512	1.732 (0.489–6.147)	0.394
**3rd** 7.09 (6.69∼7.50)	4.018 (1.444–11.177)	0.008	4.095 (1.464–11.459)	0.007	4.931 (1.617–15.037)	0.005

The highest tertile group showed a significant increased risk for an annual decline of eGFR ≥4 ml/min/1.73 m^2^/year and >25% decline in eGFR.

Abbreviations: CI, confidential interval; eGFR; estimated glomerular filtration rate.

^a^ Multivariable-adjusted odds ratio: adjusted for age, sex, systolic blood pressure, body mass index, erythrocyte sedimentation rate, fasting glucose, hemoglobin, high density lipoprotein cholesterol, triglyceride, hypertension, diabetes mellitus, smoking, exercise, estimated glomerular filtration rate.

^b^ Rapid progression in renal dysfunction was defined as a decline of eGFR ≥4 ml/min/1.73 m^2^/year.

The incidence of more than 25% decline in eGFR was seen among tertiles. The incidence of a >25% decline in eGFR gradually increased with an increase in BFP (3.8%, 5.4%, and 13.8% in lowest, middle, and highest tertiles, respectively; *P* = 0.005). The odds ratio for a >25% decline in eGFR was evaluated according to the changes in BMI and BFP. In tertiles of BFP, odds ratios were increased according to the changes of BFP. In unadjusted analysis, odds ratios were 1.423 (95% CI: 0.440–4.604) and 4.018 (95% CI: 1.444–11.177) in middle and highest tertiles, respectively, compared to the lowest tertile. Multivariate analysis also showed a gradual increase in the relative risk: 1.732 (95%CI: 0.489–6.147) and 4.931 (95% CI: 1.617–15.037) in middle and highest tertiles, respectively.

### Decline in renal function according to the increase of body fat in patients with lower GFR

**Figure 3:**
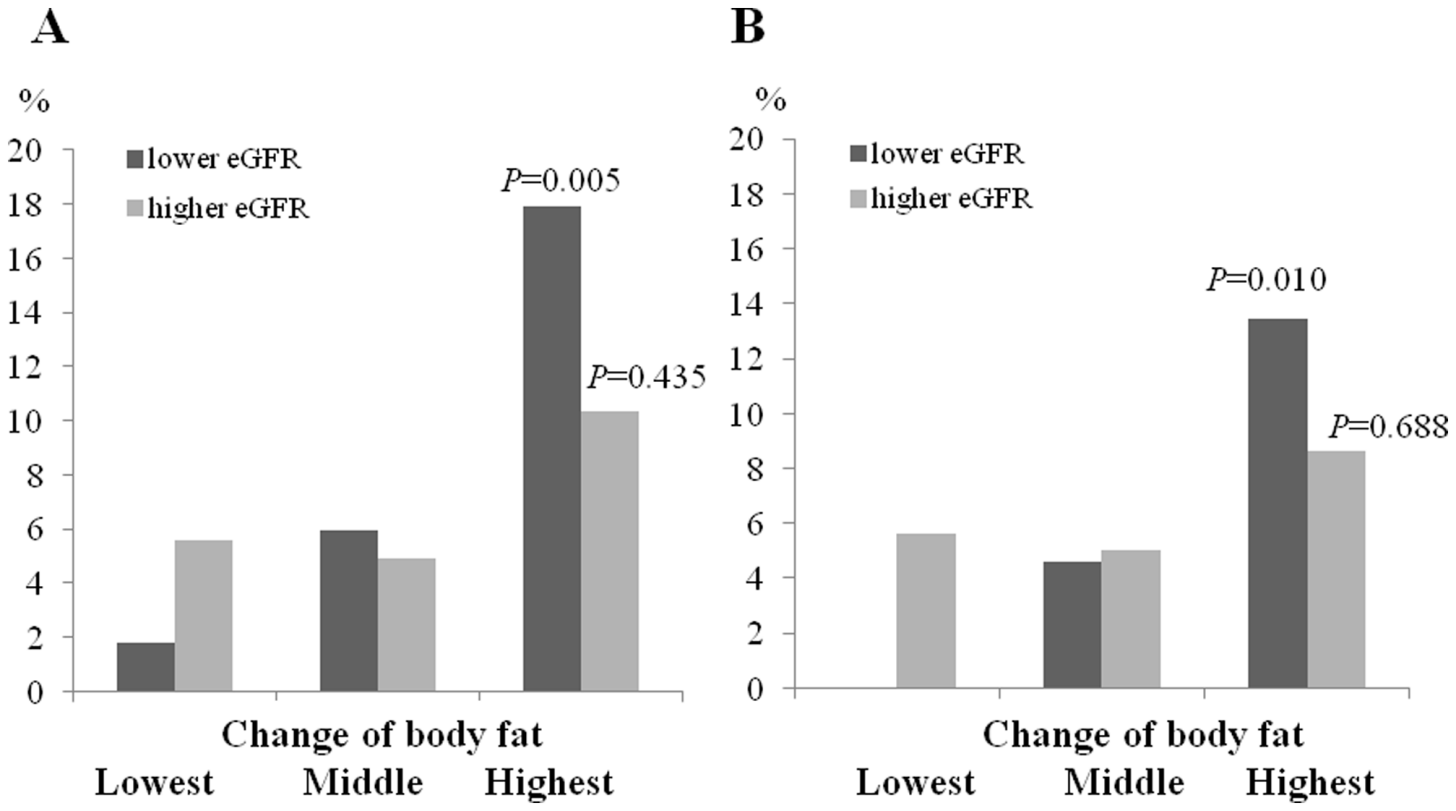
The incidence of renal outcome according to the changes of body fat. (A) The incidence of rapid renal according to the increase in body fat percent in participants with lower eGFR and higher eGFR . (B) The incidence of decline in eGFR>25% according to the increase in body fat percent in participants with lower eGFR and higher eGFR.

We divided participants into two halves according to baseline eGFR, and analyzed the prevalence of renal outcome in combination with tertiles of changes of BFP. In participants with lower eGFR, the incidence of rapid renal progression was significantly increased according to the increase in body fat percent (*P* = 0.010). However, rapid progression was not significantly increased in participants with higher eGFR (*P* = 0.435) (Figure 3A). Similarly, the incidence of decline in eGFR>25% was the highest in highest tertile of BFP in lower eGFR (*P* = 0.005). In participants with higher eGFR, the incidence of decline in eGFR>25% was not different among tertile groups (*P* = 0.688) (Figure 3B).

## Discussion

The prevalence of obesity is growing worldwide, and currently approximately 50% of the world's adult population is either obese of overweight [24], and the prevalence of obesity is a growing problem among the elderly. BFP increases significantly with age, and peak values are reached in people older than 60–70 years of age [16]–[18]. An increased in body fat is related to a decrease in energy expenditure, including resting metabolic rate, physical activity, and hormonal changes [25], [26], [27], [28], [29]. In this study, BFP increased by a median of 3.2% over the follow-up period of 5.3 years, although BMI, WC, and WHR did not change significantly. The relationship between BMI and body fat can be altered in the elderly because of the increase in body fat and loss of height, bone, and muscle.

Several studies have evaluated the use of body fat measures, estimated by BIA, in the detection of obesity, and BIA may well be better than BMI for determining the extent body fat in CKD patients [30]. In a cross-sectional study, BFP, as determined by BIA, was more effective than BMI for the detection of CVD risk factors [31]. It is known that visceral fat accumulation, estimated by BIA, is correlated with urine albumin creatinine ratio in a middle-aged Asian population [32]. However, little is known about the effect of body fat on long-term renal outcomes in the elderly.

Obesity is associated with CKD and is linked to hemodynamic and structural changes in the kidney. Anatomical changes include glomerulomegaly, increased mesangial matrix, a reduced number of podocytes, and focal segmental glomerulosclerosis [33]. These are considered to be associated with renal hemodynamic changes such as an increased renal blood flow and hyperfiltration [34]. In addition, in CKD patients, obesity is associated with inflammation and metabolic syndrome, and a high BMI is strongly associated with C-reactive protein (CRP) levels [35]. A recent study reported that the annual variation in BMI and WC is related to changes in CRP levels [36]. In that study, changes in BFP were associated with markers of inflammation, even though there were no significant differences in these markers at baseline. The indicators of metabolic syndrome were related to changes in BFP. This finding suggests that the adverse effect of BFP on renal function may well be associated with inflammation and dyslipidemia.

Another recent study showed that baseline fat mass, WC, and BMI were all related to an increased risk of rapid GFR loss (>3 ml/min/1.73 m^2^/year) in the elderly living in the United States [37]. We investigated the effect of change in BFP, BMI, and WC on several renal outcomes (data not shown). A change in BMI and WC did not result in a significant difference in any of the renal outcomes among the tertile groups. Changes in BFP were significantly associated with an annual decline in the eGFR. Participants in the highest tertile had a 4.9-fold increase in the risk for rapid progression and the development of ≥25% decline in eGFR, compared to the lowest tertile. In addition, subjects with lower baseline eGFR showed stronger impact on renal function according to the increases in BFP. The adverse effect of BFP might be more harmful in subjects with reduced renal function.

The strength of this study was that it was focused on the change in BFP. We measured BIA at baseline and then again in a follow-up study. In addition, we analyzed the longitudinal changes of renal function. Multiple renal outcomes were evaluated using changes in obesity markers. The effects of change in BFP were consistent with the annualized change in eGFR, the incidence of rapid progression in kidney dysfunction, and a >25% decline in eGFR. Finally, this study included participants aged ≥65 years, and included a randomized prospective cohort.

Our study had some limitations. Visceral fat showed the association with accelerated atherosclerosis, type 2 diabetes, and coronary artery disease on the contrary to subcutaneous fat [38]. It was recently reported that renal sinus fat was associated with blood pressure regulation and CKD [39]. In this study, the increase of body fat is related to the increased risk of renal progression but not blood pressure. Conventional BIA estimated total fat content but had a limitation to assess regional fat distribution [40]. Therefore, further studies are needed to evaluate the relationship among regional fat distribution, blood pressure, and CKD. Second, the study was susceptible to survival bias because we included participants who had repeated measures of BIA with a mean time interval of 5.3 years between measurements. Nevertheless, the excluded participants did not differ from study population with regard to baseline measurements of body fat. Third, our participants were all Korean men and women aged ≥65 years, and caution is thus needed when extrapolating the results to people of other races or to younger populations. Forth, only 15% of our study participants had CKD (baseline eGFR <60 ml/min/m^2^). Future studies in CKD patients are required. Finally, proteinuria was not measured as a quantitative method.

In conclusion, a change in BFP is associated with a decline in eGFR in the elderly. The highest tertile of change in BFP had a 4.9-fold increase in the risk for rapid progression of renal dysfunction, while the change in BFP was correlated with the development of inflammation and dyslipidemia.
